# Comparing Treatment Plan in All Locations of Esophageal Cancer: Volumetric Modulated Arc Therapy versus Intensity-Modulated Radiotherapy

**DOI:** 10.1097/MD.0000000000000750

**Published:** 2015-05-01

**Authors:** Jang-Chun Lin, Jo-Ting Tsai, Chih-Chieh Chang, Yee-Min Jen, Ming-Hsien Li, Wei-Hsiu Liu

**Affiliations:** From the Department of Radiation Oncology, Shuang Ho Hospital, Taipei Medical University (J-CL, J-TT, C-CC, Y-MJ, M-HL); Department of Radiation Oncology, Tri-Service General Hospital, National Defense, Medical Center (J-CL); Graduate Institute of Medical Sciences (W-HL); and Department of Neurological Surgery, Tri-Service General Hospital and National Defense Medical Center, Taipei, Taiwan (W-HL).

## Abstract

The aim of this study was to compare treatment plans of volumetric modulated arc therapy (VMAT) with intensity-modulated radiotherapy (IMRT) for all esophageal cancer (EC) tumor locations.

This retrospective study from July 2009 to June 2014 included 20 patients with EC who received definitive concurrent chemoradiotherapy with radiation doses >50.4 Gy. Version 9.2 of Pinnacle^3^ with SmartArc was used for treatment planning. Dosimetric quality was evaluated based on doses to several organs at risk, including the spinal cord, heart, and lung, over the same coverage of gross tumor volume.

In upper thoracic EC, the IMRT treatment plan had a lower lung mean dose (*P* = 0.0126) and lung V5 (*P* = 0.0037) compared with VMAT; both techniques had similar coverage of the planning target volumes (PTVs) (*P* = 0.3575). In middle thoracic EC, a lower lung mean dose (*P* = 0.0010) and V5 (*P* = 0.0145), but higher lung V20 (*P* = 0.0034), spinal cord Dmax (*P* = 0.0262), and heart mean dose (*P* = 0.0054), were observed for IMRT compared with VMAT; IMRT provided better PTV coverage. Patients with lower thoracic ECs had a lower lung mean dose (*P* = 0.0469) and V5 (*P* = 0.0039), but higher spinal cord Dmax (*P* = 0.0301) and heart mean dose (*P* = 0.0020), with IMRT compared with VMAT. PTV coverage was similar (*P* = 0.0858) for the 2 techniques.

IMRT provided a lower mean dose and lung V5 in upper thoracic EC compared with VMAT, but exhibited different advantages and disadvantages in patients with middle or lower thoracic ECs. Thus, choosing different techniques for different EC locations is warranted.

## INTRODUCTION

Esophageal cancer (EC) remains one of the most aggressive and lethal digestive diseases worldwide. It is associated with poor outcomes and presents a challenge to surgeons, doctors, and radiation oncologists. There are approximately 16,000 newly diagnosed patients with EC each year, and an estimated 14,000 patient deaths were reported in the United States in 2008.^1^ Squamous cell carcinoma is commonly seen in Asian countries, whereas adenocarcinoma is common in Europe and America. Most EC patients are at an advanced stage or are unresectable at the time of initial diagnosis.^2^ Concurrent chemoradiotherapy (CCRT) is the major treatment method for local advanced or unresectable esophageal cancer, but the 5-year overall survival rate is only 15% to 25%.^3^ Local failure is the most common failure pattern associated with CCRT, and local persistence of the disease occurs in 60% to 70% of patients.^4^ The findings of this study indicate that radiation dose escalation may improve their prognosis.

The results of the Radiation Therapy Oncology Group 94-05 trial demonstrated few survival benefits for the group receiving a higher dose of radiation therapy.^5^ However, the investigators used a traditional 2-dimensional (2D) technique with anteroposterior/posteroanterior (AP/PA) field arrangement to deliver radiotherapy (RT), which limited the dose provided to the tumor because of concerns about the safety of the surrounding healthy tissue. Studies have shown that the use of modern RT techniques is needed to clarify the possible benefits of dose escalation. Intensity-modulated radiotherapy (IMRT) constitutes an important advance in techniques for improving tumor coverage and reducing the doses delivered to the surrounding normal tissues. IMRT is superior to 3D conformal radiotherapy (3D-CRT) or 2D-RT based on dosimetric analysis.^6,7^ Volumetric modulated arc therapy (VMAT), a novel form of IMRT that was first proposed by Yu in 1995,^8^ is a widely used radiation technique and is regarded as a new generation linear-accelerator IMRT. VMAT can promote the delivery of a substantial radiation dose to the tumor while avoiding the delivery of an excess dose to the healthy tissues in the tumor vicinity. Moreover, VMAT can produce plans that are dosimetrically equivalent to IMRT for centrally located cancers such as cancers of the anal canal, prostate cancer, cervical cancer, and head and neck cancers.^9–11^ Therefore, the evaluation of the efficiencies and dosimetric distributions of VMAT in comparison with IMRT should be elucidated.

In this study, we compared VMAT and conventional IMRT for patients with EC in all locations with respect to the dose distributions, planning target volumes (PTVs), and organs at risk (OARs).

## MATERIALS AND METHODS

### Patient Data and Simulation

Patients were treated for primary tumors or regional lymph node metastases using methods approved by the multidisciplinary thoracic tumor board at Shuang Ho Hospital. All procedures of patient acquisition followed the tenets of the Declaration of Helsinki and were approved by the Institutional Review Committee at Shuang Ho Hospital, Taipei Medical University. Patients previously treated at our facility for EC at any location were chosen for this study. The patient inclusion criteria included an age of 20 to 80 years and an Eastern Cooperative Oncology Group (ECOG) performance score of 0, 1, or 2. Tumors were staged according to the 6th edition of the American Joint Committee on Cancer (AJCC) using the 2006 Criteria and the 7th edition of the AJCC using the 2010 Criteria. Positron emission tomography (PET) or computed tomography (CT) was used to rule out the existence of distant metastases.

CT images without intravenous contrast of simulation were acquired with the patient in the supine position and immobilized by gripping the overhead arm positioner (Medtec and Sinmed Radiation Oncology Products, Orange City, IA) over the patient's head. The skin line marker was set at a slice thickness of 3 to 5 mm. A gross tumor volume (GTV) including the gross esophageal tumor and positive regional lymph nodes was contoured by a physician based on the PET fusion image. The clinical targeted volume (CTV) was defined as the GTV plus 3 to 5 mm to the anterior, posterior, right, and left directions and 5 cm into the superior and inferior regions. PTV margins were provided by the physician and varied from case to case. The prescription dose was 1.8 Gy × 28 fractions for a total dose of 50.4 Gy. OARs included the heart, lungs, spinal cord, stomach, and kidneys.

All plans aimed to achieve a minimum dose >95% and a maximum dose <110% of the prescribed dose. The primary objectives with regard to the OARs were defined as follows: spinal cord Dmax <45 Gy; and lungV20 <35%, V10 <45% and V5 <65%. The secondary objectives were as follows: mean dose of lung <20 Gy; heart V40 ≤50%; and mean dose of heart <26 Gy. As a result of the tumor coverage requirements, a waiver could be applied for these dose constraints. Vx means the percentage of organ receiving more or equal to x Gy.

### VMAT Technique

For treatment planning, images were acquired using a spiral CT scanner without contrast. The VMAT plans used 2 to 4 partial arcs sharing the same isocenter. The treatment protocols for the 9 patients treated with 2 partial arcs were planned with start and stop angles of 150 and 211 degrees, respectively, that were delivered with a counterclockwise rotation. The protocols for the remaining 11 patients were planned with 4 partial arcs. The first and second of these arcs rotated from 181 to 340 degrees with clockwise and counterclockwise rotation, whereas the third and fourth partial arcs rotated from 41 to 180 degrees with clockwise and counterclockwise rotation.

### IMRT Technique

A 15-MV photon beam with 5 to 6 co-planar beams and CT-based treatment planning (Pinnacle version 9.2) was used. The doses were delivered using a linear accelerator (LINAC) equipped with multileaf collimators (MLCa). Similar coverages of CTV compared with IMRT and VMAT were confirmed.

### Statistical Analysis

Data were collected retrospectively from medical records, and 20 patients were included in this analysis. The differences in the dosimetric parameters between the 2 planning techniques were evaluated using Wilcoxon's signed-rank test. Data analysis was performed using the Statistical Package for Social Sciences (SPSS) 17 (SPSS Inc, Chicago, IL). A *P* value <0.05 was considered statistically significant.

## RESULTS

### Patient Characteristics

Twenty patients (18 males and 2 females) previously treated at our facility for 6 upper thoracic, 8 middle thoracic, and 6 lower thoracic ECs were chosen for this study. All patients were diagnosed with moderately to poorly differentiated squamous cell carcinomas of the esophagus. Five patients were stage IIB, 6 were stage IIIA, 3 were stage IIIB, and 6 were stage IIIC according to the 6th edition of the AJCC, 2006 Criteria, and the 7th edition of the AJCC, 2010 Criteria. All patients in this study received concurrent chemotherapy. Table 1 summarizes the patients’ characteristics.

**TABLE 1:**
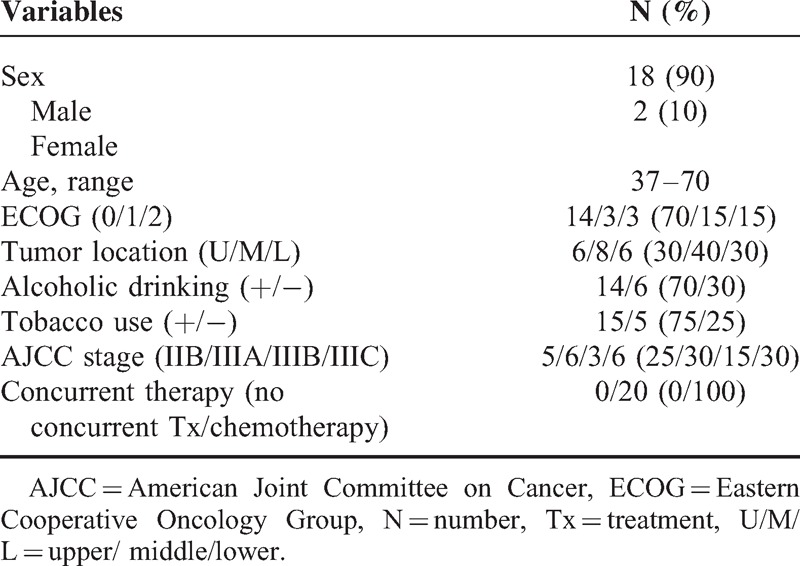
Patients and Tumor Characteristics (N = 20)

Planning dosimetry of the 20 patients receiving VMAT and IMRT was analyzed regardless of the tumor location. Similar PTV coverage (*P* = 0.2685) and V10 of the lung (*P* = 0.1650) were found. VMAT had lower spinal cord Dmax (*P* = 0.0389), heart mean dose (*P* = 0.0002), and V20 of the lung (0.0090) values compared with IMRT. In contrast, the IMRT for EC was superior to VMAT in V5 of the lung (*P* < 0.0001) and the lung mean dose (*P* < 0.0001). Different treatment planning had a borderline effect on monitor units (MUs) (*P* = 0.0839). All dosimetric results for PTV and MUs and the comparison of OARs in all EC cases are detailed in Table 2; these data were analyzed using Wilcoxon signed-rank test.

**TABLE 2:**
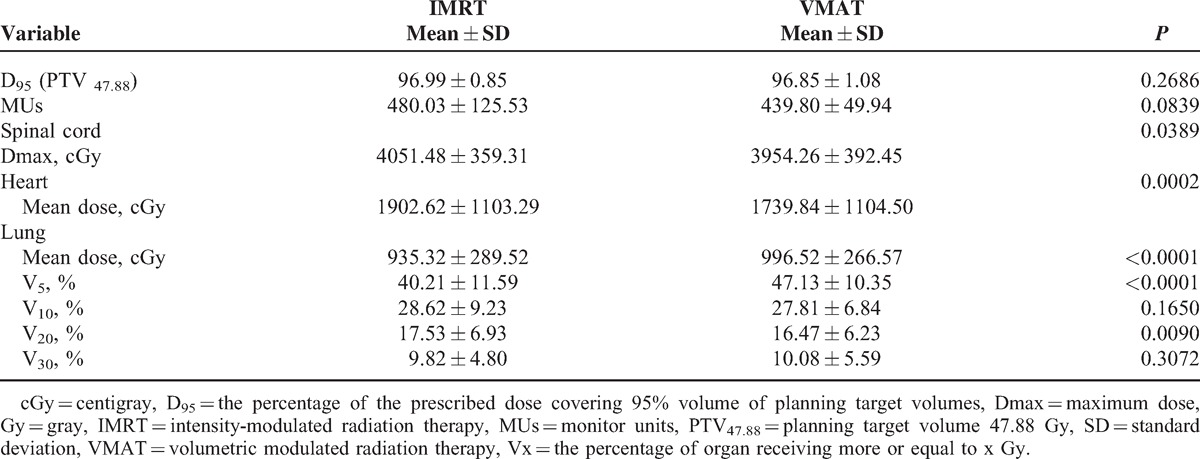
Dosimetric Results for Planning Target Volume and MUs and Comparison for Organs At Risk in All Locations of Esophageal Cancer

**FIGURE 1:**
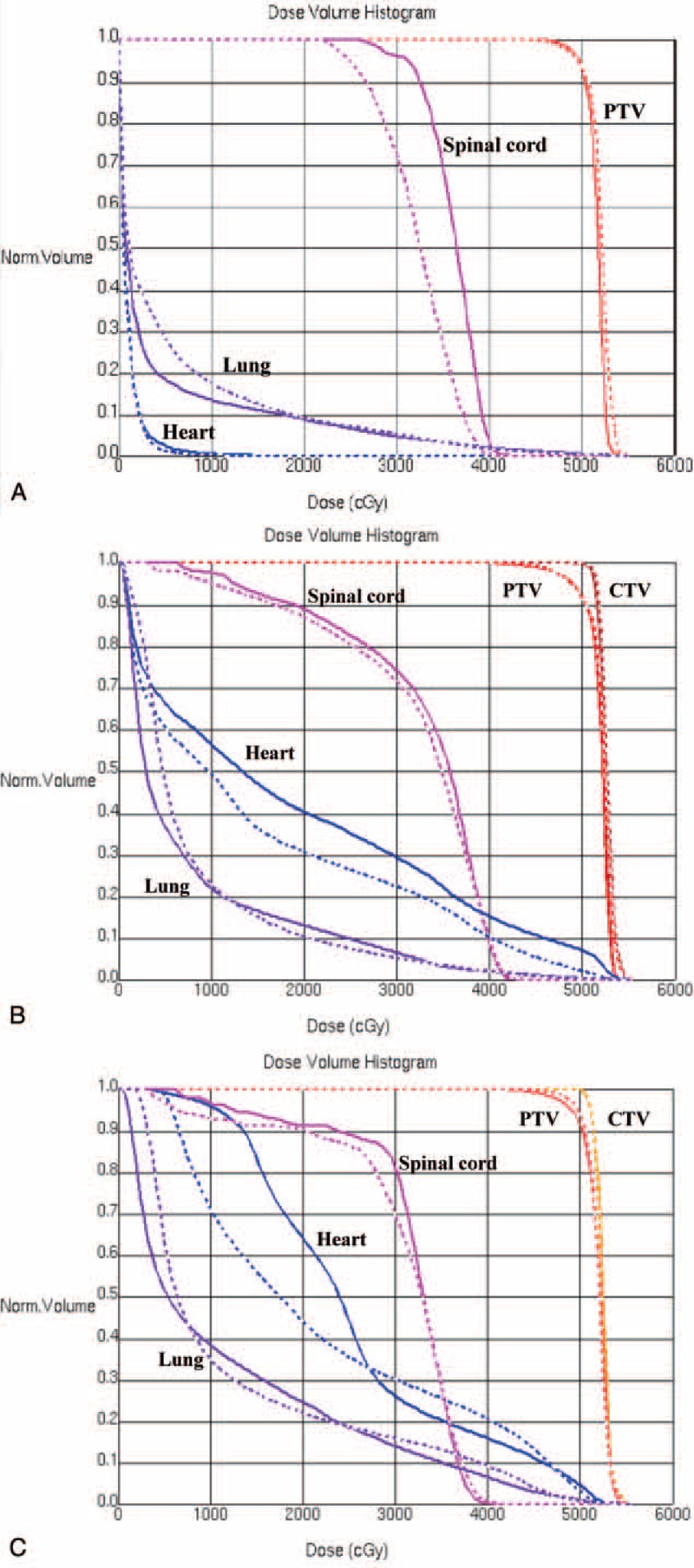
(A) Comparing the dose–volume histogram from VMAT and IMRT of a patient with upper third esophageal tumor. Dashed line: VMAT; solid line: IMRT. (B) Comparing the dose–volume histogram from VMAT and IMRT of a patient with middle third esophageal tumor. Dashed line: VMAT; solid line: IMRT. (C) Comparing the dose–volume histogram from VMAT and IMRT of a patient with lower third esophageal tumor. Dashed line: VMAT; solid line: IMRT. IMRT = intensity modulated radiation therapy, VMAT = volumetric modulated radiation therapy.

Further statistical analysis was conducted based on the different locations of the EC. In upper thoracic EC, the IMRT treatment plan exhibited a lower lung mean dose (*P* = 0.0126) and lung V5 (*P* = 0.0037) compared with VMAT, and a similar coverage of PTV (*P* = 0.3575). Figure 1A depicts the dose distribution of IMRT and VMAT in a patient with upper EC. Figure 2  Adisplays the dose-volume histograms (DVHs) for the 2 different plans in a typical case, and Table 3 summarizes other dosimetric results in detail.

**FIGURE 2:**
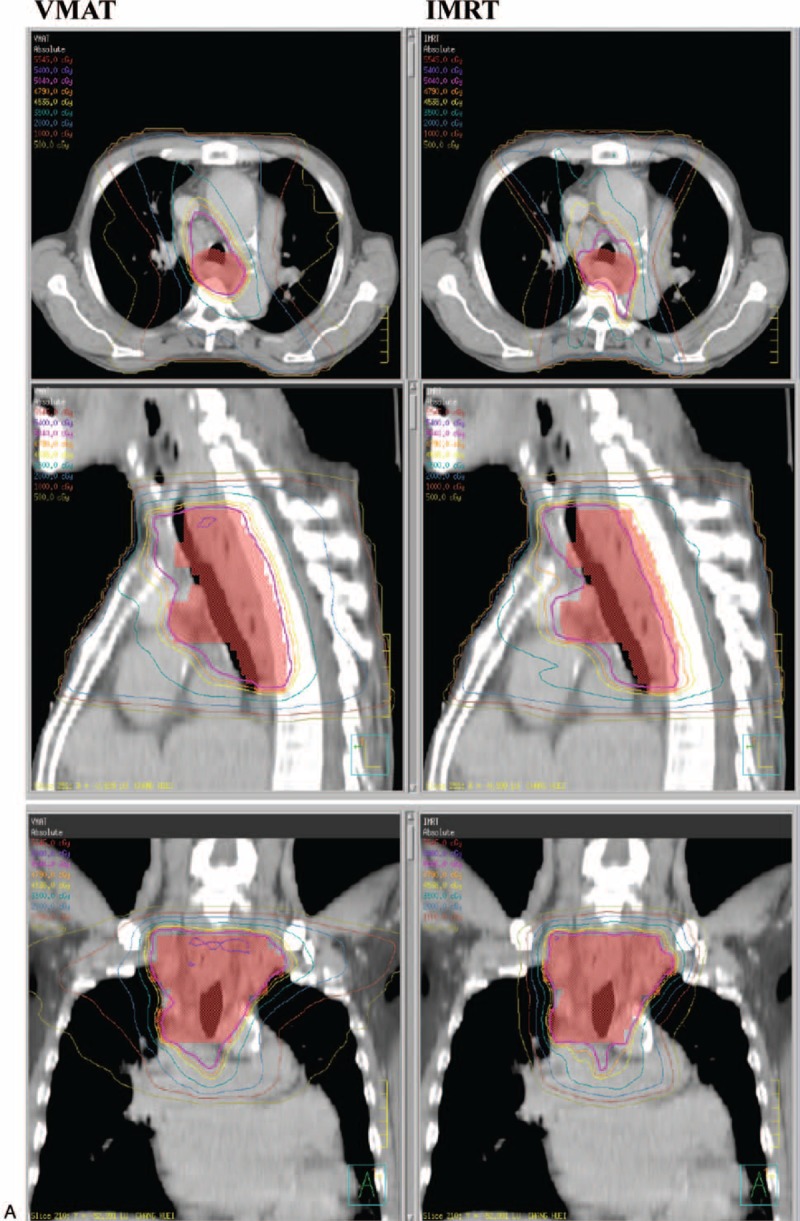
(A) Dose distributions of VMAT (left) and IMRT (right) for a upper third esophageal cancer in axial, sagittal, and coronal views. (B) Dose distributions of VMAT (left) and IMRT (right) for a middle third esophageal cancer in axial, sagittal, and coronal views. (C) Dose distributions of VMAT (left) and IMRT (right) for a lower third esophageal cancer in axial, sagittal, and coronal views. IMRT = intensity modulated radiation therapy, VMAT = volumetric modulated radiation therapy.

**FIGURE 2 (Continued):**
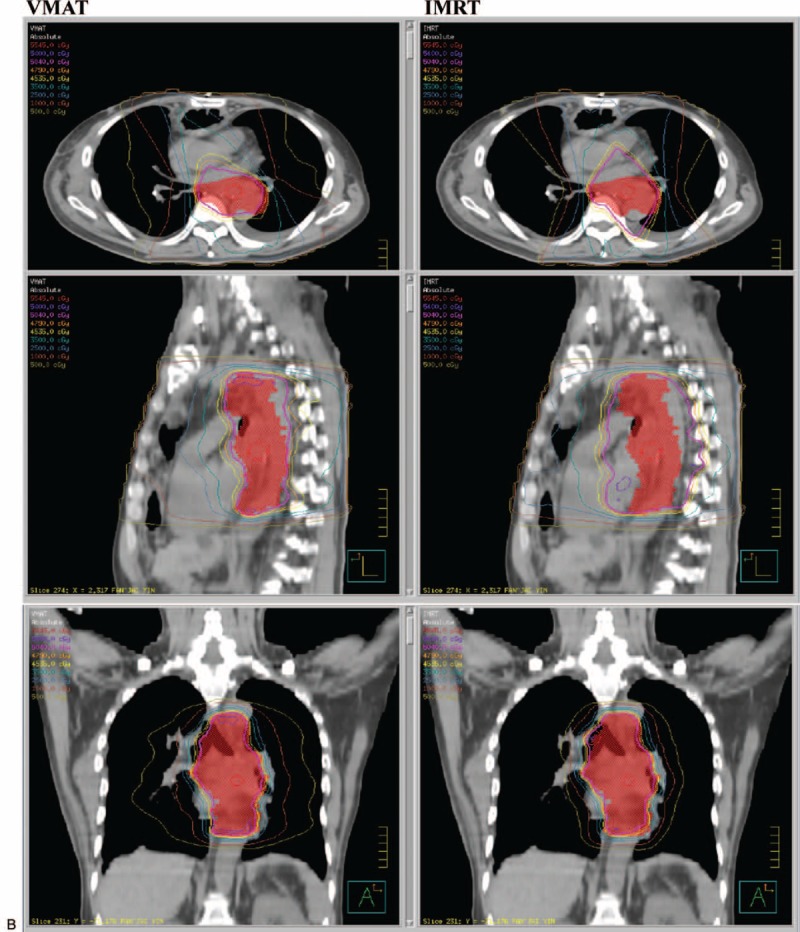
(A) Dose distributions of VMAT (left) and IMRT (right) for a upper third esophageal cancer in axial, sagittal, and coronal views. (B) Dose distributions of VMAT (left) and IMRT (right) for a middle third esophageal cancer in axial, sagittal, and coronal views. (C) Dose distributions of VMAT (left) and IMRT (right) for a lower third esophageal cancer in axial, sagittal, and coronal views. IMRT = intensity modulated radiation therapy, VMAT = volumetric modulated radiation therapy.

**FIGURE 2 (Continued):**
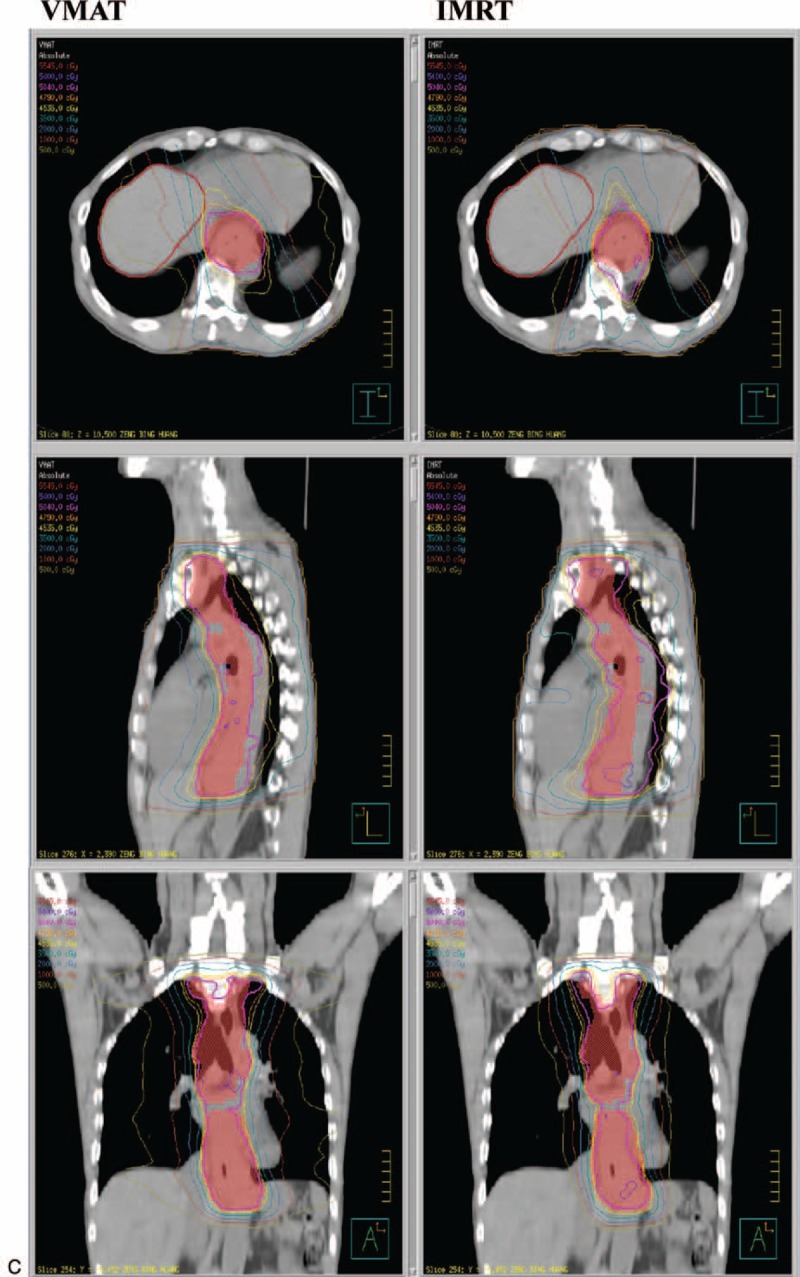
(A) Dose distributions of VMAT (left) and IMRT (right) for a upper third esophageal cancer in axial, sagittal, and coronal views. (B) Dose distributions of VMAT (left) and IMRT (right) for a middle third esophageal cancer in axial, sagittal, and coronal views. (C) Dose distributions of VMAT (left) and IMRT (right) for a lower third esophageal cancer in axial, sagittal, and coronal views. IMRT = intensity modulated radiation therapy, VMAT = volumetric modulated radiation therapy.

**TABLE 3:**
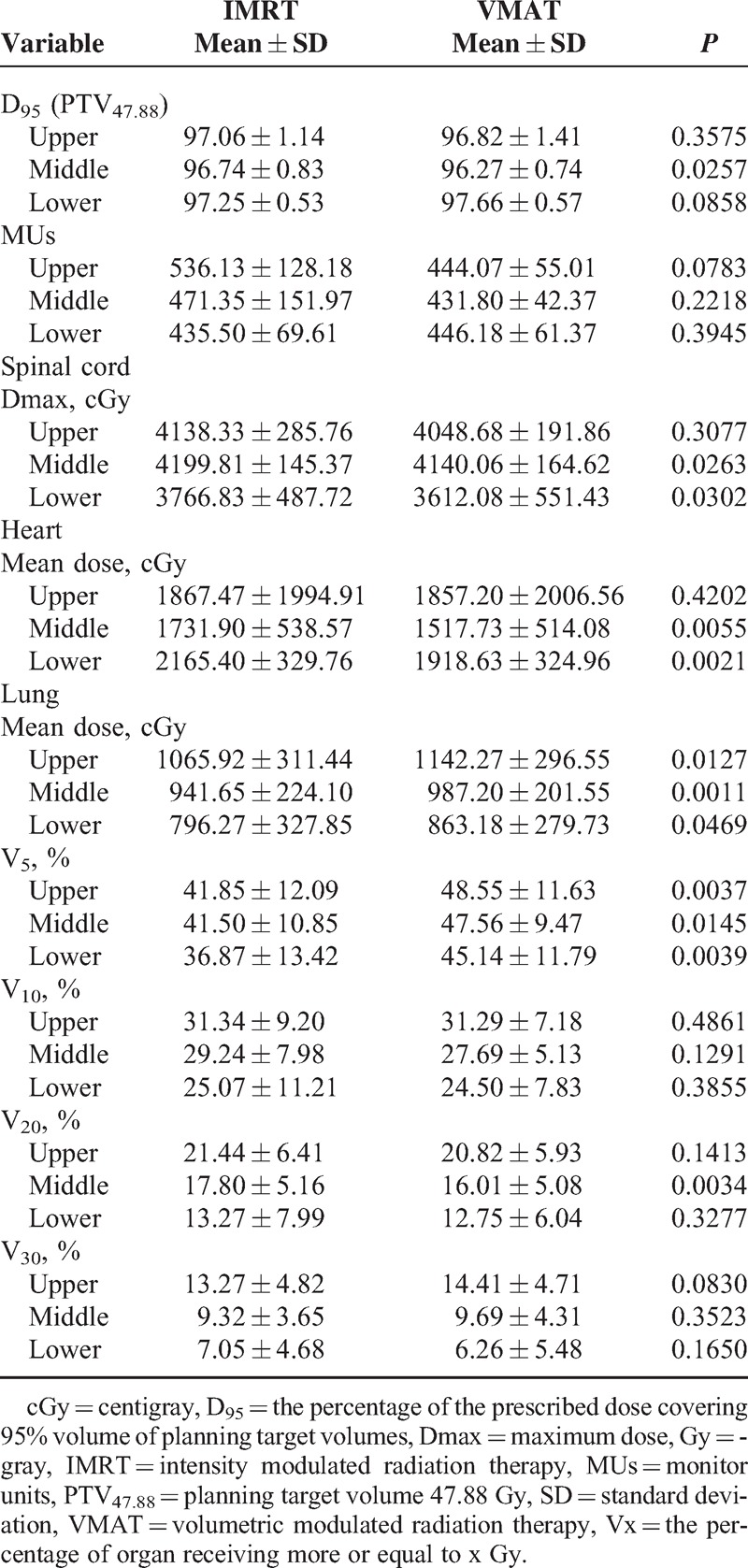
Dosimetric Results for Planning Target Volume and MUs and Comparison for Organs At Risk in Upper, Middle, and Lower Thoracic Esophageal Cancer

IMRT was characterized by a lower lung mean dose (*P* = 0.0010) and V5 (*P* = 0.0145), but a higher lung V20 (*P* = 0.0034), spinal cord Dmax (*P* = 0.0262), and heart mean dose (*P* = 0.0054), compared with VMAT in patients with middle thoracic EC; additionally, IMRT provided better coverage of PTV. The dose distribution of IMRT and VMAT in 1 middle EC patient and the DVHs for the 2 different plans in a typical case are presented in Figure 1B and Figure 2  B. The patients with lower thoracic EC had a lower lung mean dose (*P* = 0.0469) and V5 (p = 0.0039), but a higher spinal cord Dmax (p = 0.0301) and heart mean dose (*P* = 0.0020), following IMRT compared with VMAT. The PTV coverage was similar (*P* = 0.0858) for the 2 techniques. The DVHs and dose distributions of IMRT and VMAT in 1 patient with lower EC are displayed in Figure 1C and Figure 2  C.

## DISCUSSION

Previous studies^12,13^ have demonstrated the use of VMAT at dosages ranging from 50.4 to 60 Gy for EC, but the feasibility of the high-dose VMAT technique has not yet been demonstrated. In the present study, IMRT provided better OAR dose sparing (eg, lung mean dose and V5 of the lung) compared with VMAT in EC patients regardless of the tumor location. However, IMRT provided higher OAR doses (V20 of the lung, spinal cord Dmax, and heart mean dose) compared with VMAT in patients with middle and lower thoracic EC. Moreover, IMRT had equivalent PTV coverage with VMAT in patients with upper and lower thoracic EC but better PTV coverage in patients with middle thoracic EC. Our study presented a dosimetric comparison in patients with EC tumors in all locations. The results demonstrated that IMRT could generate better radiotherapeutic plans than VMAT only in patients with upperthoracic EC.

The difficulty of ensuring that healthy tissues receive low doses in patients who require increasing volumes has been demonstrated with comparisons between IMRT and 3D-CRT.^14^ IMRT is capable of better conforming higher doses to the treatment volume compared with 3D-CRT. The increased number of beams improved conformality, and a greater volume of healthy tissue received the dose. The ability to edit beam fluences should be considered an important difference between VMAT and IMRT. Dosimetry can edit fluences when planning IMRT, but not when planning VMAT, in the Eclipse Treatment Planning System.

The initial commercial use of VMAT planning and technology was developed in 2008, but the use of the technique has increased rapidly. VMAT is a complex form of IMRT that provides dose delivery in single or multiple arcs. As shown by a number of studies,^15,16^ 2 arcs provide better modulation factors during optimization due to the capacity for independent optimization, dose rate, and gantry speed combinations; therefore, the delivery time can be decreased. Previous studies reported some of the advantages of VMAT compared with IMRT.^10,17^ For example, Tsai et al^17^ compared VMAT plans with IMRT plans and found that VMAT plans presented a significantly shorter delivery time. VMAT provided adequate sparing of OARs and coverage of PTV that were at least equivalent to IMRT; additionally, it could significantly decrease the number of MUs and the treatment time required for the morbidities.^18^ The biological advantage of the shorter delivery time of the VMAT technique is based on cancer cell killing and, thus, may result in good local disease control. Moreover, the advantage of the delivery of lower MUs resulted in a lower dose to normal tissue and a reduced probability of the development of secondary cancer.^19^ In our study, VMAT was not consistently superior to IMRT in the sparing of organs at risk or in PTV coverage; however, this technique was very successful in decreasing the number of MUs required for the treatment of ECs in upper and middle thoracic EC, and increasing MU in lower thoracic EC. Although there was no statistical significance, Yin et al's^13^ 2012 study compared the conventional sliding window IMRT plans with VMAT plans in EC. This study indicated that the V20 of the lung and the lung mean dose were important predictors for radiation pneumonitis (RP). Moreover, the authors suggested that the V20 of the lung and lung mean dose were important DVH factors for RP based on the analysis of normal tissue effects in the clinic.^20^ However, Marks et al^21^ suggested limiting the V20 to ≤30% to 35% and the lung mean dose to 20 to 23 Gy to reduce the dose–volume effect in the lung. Therefore, our study limited the V20 of normal lung tissues to ≤35% and the lung mean dose to ≤20 Gy. Furthermore, Wang et al revealed that the V5 of the lung was the most important predictor for RP.^22^ In the present study, we found that the V5 maybe the best predictor for RP in patients with a history of smoking. Another study showed that RP never occurred below a single dose of 7 Gy to the whole lung.^23^ Our results demonstrated that the V20 of the lung and the lung mean dose are more critical for predicting lung problems.

In conclusion, VMAT was not always superior to IMRT in sparing the organs at risk or in PTV coverage during treatment of EC. However, VMAT offered an equivalent or better dose sparing of the lung and heart and a significant reduction in MUs per fraction. For upper EC patients, the PTV was T-shaped across the chest and neck; in these patients, VMAT provided a fairly uniform dose distribution. For patients with middle and lower EC in which the PTV involved more of the lung tissue, VMAT treatment had the potential to significantly increase the coverage of the lungs at low doses and the most uniform dose distribution. Compared with VMAT, IMRT provided a lower mean dose and V5 of the lungs in patients with upper thoracic EC, but exhibited different advantages and disadvantages in patients with middle or lower thoracic EC.
